# The development of a solid lipid nanoparticle (SLN)-based lacticin 3147 hydrogel for the treatment of wound infections

**DOI:** 10.1007/s13346-023-01332-9

**Published:** 2023-03-24

**Authors:** Aoibhín Ryan, Pratikkumar Patel, Poonam Ratrey, Paula M. O’Connor, Julie O’Sullivan, R. Paul Ross, Colin Hill, Sarah P. Hudson

**Affiliations:** 1 Department of Chemical Sciences, Bernal Institute, University of Limerick, Limerick, Ireland; 2 Teagasc Food Research Centre, Moorepark, Fermoy, Co. Cork Ireland; 3 APC Microbiome Ireland, Cork, Ireland; 4 School of Microbiology, University College Cork, College Road, Cork, Ireland; 5 SSPC the SFI Research Centre for Pharmaceuticals, University of Limerick, Limerick, Ireland

**Keywords:** Lacticin 3147, Bacteriocins, Hydrogels, Solid lipid nanoparticles, Drug delivery, Antimicrobial resistance

## Abstract

Chronic wounds affect millions of people globally. This number is set to rise with the increasing incidence of antimicrobial-resistant bacterial infections, such as methicillin-resistant *Staphylococcus aureus* (MRSA), which impair the healing of chronic wounds. Lacticin 3147 is a two-peptide chain bacteriocin produced by *Lactococcus lactis* that is active against *S. aureus* including MRSA strains. Previously, poor physicochemical properties of the peptides were overcome by the encapsulation of lacticin 3147 into solid lipid nanoparticles. Here, a lacticin 3147 solid lipid nanoparticle gel is proposed as a topical treatment for *S. aureus* and MRSA wound infections. Initially, lacticin 3147’s antimicrobial activity against *S. aureus* was determined before encapsulation into solid lipid nanoparticles. An optimised gel formulation with the desired physicochemical properties for topical application was developed, and the lacticin-loaded solid lipid nanoparticles and free lacticin 3147 aqueous solution were incorporated into separate gels. The release of lacticin 3147 from both the solid lipid nanoparticle and free lacticin gels was measured where the solid lipid nanoparticle gel exhibited increased activity for a longer period (11 days) compared to the free lacticin gel (9 days). Both gels displayed potent activity ex vivo against *S. aureus-*infected pig skin with significant bacterial eradication (> 75%) after 1 h. Thus, a long-acting potent lacticin 3147 solid lipid nanoparticle gel with the required physicochemical properties for topical delivery of lacticin 3147 to the skin for the potential treatment of *S. aureus*-infected chronic wounds was developed.

**Graphical abstract:**

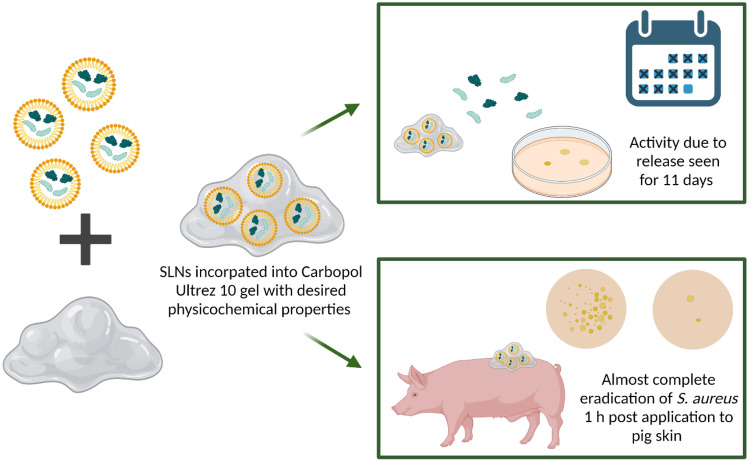

**Supplementary Information:**

The online version contains supplementary material available at 10.1007/s13346-023-01332-9.

## Introduction

With the impending antimicrobial resistance crisis, alternatives to traditional antibiotics for the treatment of drug-resistant infections are urgently required [1, 2]. Bacteriocins are one alternative being investigated [3]. Bacteriocins are antimicrobial peptides ribosomally produced by bacteria [4]. Their poor physicochemical properties, however, are one of the factors which have limited their medical application as antimicrobials [5]. Lacticin 3147 is a dual-acting bacteriocin produced by *Lactococcus lactis* which is active against many clinically relevant and antimicrobial-resistant Gram-positive bacteria [6–8]. Its two peptides, Ltnα and Ltnβ, act synergistically, inhibiting peptidoglycan synthesis and forming pores in the cell membrane of the target bacterium [9, 10]. The lacticin 3147 peptides have been shown previously to be degraded by pancreatic proteases and have poor aqueous solubility and stability [11]. These limitations can be overcome, however, by the encapsulation of lacticin 3147 into solid lipid nanoparticles (SLNs) where the SLN formulation exhibited prolonged activity against *L. monocytogenes* as well as resistance to enzymatic degradation [11, 12].

Chronic wounds are a serious healthcare issue affecting millions of patients globally. They affect 1–2% of the population in developed countries [13, 14], with an estimated healthcare cost of 20 billion dollars per annum in the US alone [15, 16]. The prevalence rate of chronic wounds equates to that of heart failure in such countries [15, 17]. One of the main factors which affect the healing process of chronic wounds is superficial infections in the wound [18, 19], which are mostly caused by the bacteria *Pseudomonas aeruginosa* and *Staphylococcus aureus* [20]. With antimicrobial-resistant bacterial wound infections such as methicillin-resistant *S. aureus* (MRSA) infections becoming more prevalent, the treatment of wound infections is getting even tougher [21, 22]. The recommended treatment of bacterial wound infections usually involves parenteral or oral antibiotics [22]. Due to the systemic nature of these drug administration routes, a higher dosage is required to ensure the required concentration of the drug arrives at the wound site [22], thereby increasing side effects and promoting antimicrobial resistance. Thus, a topical formulation of lacticin 3147 administered locally could be an alternative treatment for wound and chronic wound infections. It would allow for potent antimicrobial activity at the site of action, at lower administered doses, without the pitfalls of systemically delivered drugs [23]. The formulation could also be used against antimicrobial-resistant bacteria due to lacticin 3147’s activity against such bacterial strains [8, 24]. A topical lacticin 3147 SLN hydrogel formulation could also allow for the sustained release of active peptides thus preventing bacterial growth over time [25].

SLNs can be used as drug carriers for dermal and topical use [26]. As previously shown, SLNs can encapsulate hydrophobic drugs, like lacticin 3147, increasing their solubility, stability and activity in aqueous solutions [11]. SLNs can also act as occlusives leading to increased hydration and, in turn, can improve skin permeability [27–29]. This is due to the formation of a mono-layered film of drug-containing nanoparticles, following evaporation of water from the SLN formulation, which reduces water loss from the skin. They also enhance the stability of the drugs and modulate their release. SLNs can be applied to inflamed or damaged skin due to their non-irritant and nontoxic constituents [30, 31]. Another advantage of SLNs for dermal and topical use is the protection they provide to the encapsulated drug against degradation [32, 33]. To create a formulation that allows for easy application to the skin, SLN dispersions are commonly incorporated into a suitable base such as a gel (Table 1) [26, 34].

**Table 1:** Topical and dermal drug delivery by SLN gels

**Drug**	**SLN formulation**	**Gelling agent**	**Purpose**	**Advantages**
Aceclofenac [35]	Glyceryl monostearate, lecithin, Tween 80	Carbopol^®^ 934	Anti-inflammatory—rheumatoid arthritis	Prolonged inhibition of oedema and skin targeting compared to free drug gel
Meloxicam [36]	Cetyl palmitate, propylene glycol, Tween 80	Carbopol^®^ 940	Anti-inflammatory—rheumatoid arthritis	Prolonged release and oedema inhibition, and enhanced skin retention and delivery compared to nanoemulsion gel
Piroxicam [37]	Brij 35, Brij 72, cholesterol, stearic acid	Carbopol^®^ 934	Anti-Inflammatory—rheumatoid arthritis	Enhanced skin permeation of drug over commercial piroxicam gel
Resveratrol [38]	Precirol^®^ ATO 5, Tween 20, Span 80	Carbopol^®^ 940	Anti-inflammatory- contact dermatitis	Enhance skin layer targeting and oedema reduction compared to free drug gel with no irritation
Silybin [39]	Precirol^®^ ATO5, Tween 20, Span 80	Carbopol^®^ 940	Anti-inflammatory- contact dermatitis	Prolonged release and higher efficacy than free drug gel with no irritation
Halobetasol propionate [40]	Glyceryl monostearate, Tween 80	Carbopol^®^ 974	Anti-inflammatory- atopic and contact dermatitis	Better skin targeting and less irritation compared to plain free drug gel and commercial formulation
Fluconazole [41]	Compritol^®^ 888 ATO, poloxamer^®^ 407, chremophor RH40	Carbopol^®^ 934	Fungal infections—*pityriasis versicolor*	Enhanced therapeutic response compared to marketed Candistan cream
Eugenol [42]	Compritol^®^ 888 ATO, poloxamer^®^ 188, stearic acid	Carbopol^®^ 940	Fungal skin infections	Significantly enhanced epidermal targeting than eugenol–hydroxypropyl-β–cyclodextrin complex hydrogel and almond oil eugenol solution
Griseofulvin [43]	Tween 80, Compritol^®^ 888 ATO, Phospholipon 90G	Carbopol^®^ 980	Fungal skin infections	Increased permeation compared to conventional cream base
Miconazole nitrate [44]	Soya lecithin, tristearin, Tween 80	Carbopol^®^ 934	Fungal skin infections	Significantly greater skin retention and anti-fungal activity in vivo when compared to free drug suspension and free drug gel
Adapalene [45]	Glyceryl monostearate, pluronic F68	Carbopol^®^ Ultrez 10	Anti-acne	Enhanced dermal bioavailability anti-acne efficacy and skin tolerability compared to free drug gel
Benzoyl peroxide [46]	Precirol^®^ ATO 5, Tween 80	Carbopol^®^ 934	Anti-acne	Enhanced drug deposition, reduced skin irritation and strong anti-bacterial activity compared to marketed drug
Articaine [47]	Glyceryl tripalmitate, polyvinyl alcohol	Aristoflex^®^ AVC	Local anaesthetic	Enhanced chemical stability of the drug, increased cell viability and prolonged anaesthetic effect compared to free drug
Hydro-quinone [48]	Precirol^®^ ATO 5, poloxamer^®^ 407, span 20	Carbopol^®^ 934	Hyperpigmentation	Enhanced stability and skin penetration of drug, reduced systemic absorption compared to free drug gel
Metformin [49]	Tween 60, cholesterol, span 60, beeswax	Carbopol^®^	Skin ageing prevention	Enhanced skin delivery and skin permeation compared to free drug gel
Retinyl palmitate [50]	Precirol^®^ ATO5, Gelucire^®^ 50/13, dicetyl phosphate	Carbopol^®^ 940	Anti-wrinkle	More effective treatment compared to commercial product
Coenzyme Q10 [51]	Compritol^®^ 888 ATO, poloxamer^®^ 188, Tween 80	Carbopol^®^ 974	Antioxidant	Enhanced (×2) dermal delivery compared to free drug gel

For topical delivery to the skin, gels are said to be more user-friendly than ointments as they are less greasy and sticky when applied to the skin [52]. Hydrogels are an easy-to-apply, attractive formulation strategy for the topical cutaneous delivery of biological drugs in a controlled manner [53, 54]. They are a water-swollen, cross-linked network of polymers that form a 3D structure [55–57]. Since the discovery and use of hydrogels in the field of biomedicine in the early 1950s [58], an enormous number of hydrogels have been created and are being researched for a variety of biomedical and pharmacological uses [57]. A selection of hydrogels that have also been used in combination with SLNs are listed in Table 1. While solid lipid nanoparticles are well established as suitable carriers for peptide drugs [59], their use for the delivery of bacteriocins has been limited to well-known bacteriocins such as nisin [5] and lacticin 3147 [11]. To the best of our knowledge, the SLN-hydrogel combinatorial approach has not been used for peptide drugs to date and the examples given in Table 1 are for small molecule drugs only.

As noted in Table 1, SLN gels have been formulated mostly using the Carbopol^®^ family as gelling agents. Cellulose derivatives, such as hydroxypropylmethylcellulose (HPMC), are also commonly used gelling agents [60]. SLN gels have also shown many advantages over free drug gels and/or commercial products such as enhanced/prolonged activity, skin targeting and skin retention (Table 1). The combination of the lipid matrix of SLNs and the small size of SLNs ensuring close contact with the stratum corneum results in a high accumulation of the active agent in the skin and reduced adsorption into systemic circulation [28, 61–64]. Because of this, SLNs have been claimed to be more advantageous over other colloidal carriers for dermal drug delivery [48].

A lacticin 3147 SLN-based gel (SLNαβ gel) and a free lacticin 3147 hydrogel (freeαβ hydrogel) were produced for topical delivery for the eradication of an *S. aureus* infection on the skin. Their antimicrobial activity and release were investigated to determine if the SLNs would increase the activity of the lacticin 3147 gel or prolong the release of lacticin 3147 from the gel. This study demonstrates the potential of the delivery system for lacticin 3147 as a treatment for *S. aureus*-infected wounds.

## Methods and materials

### Materials

*L. lactis* DPC6577, a lacticin 3147-producing strain, and pig skin were provided by Teagasc Food Research Centre Moorepark, Cork, Ireland. *Listeria monocytogenes* culture (ATCC 1916) was purchased from ATCC. *S. aureus* DSMZ20231 was purchased from DSMZ, the German collection of microorganisms and cell cultures. *S. aureus Xen-29* was supplied by our collaborators in APC Microbiome Ireland, Cork. Brain heart infusion (BHI) broth, BHI agar, bovine serum albumin (BSA), dimethyl sulfoxide (DMSO), ethanol (EtOH), hydroxypropyl methylcellulose (HPMC with a molecular weight of 5.5 kDa), glutaraldehyde, phosphate-buffered saline (PBS), sodium sulphate, triethanolamine (TEA), tryptic soy agar (TSA) and tryptic soy broth (TSB) were purchased from Sigma-Aldrich Ireland. Isopropanol (IPA, > 99.9%) and yeast extract were purchased from Fisher Scientific Ireland Ltd. Fasted state simulated intestinal fluid (FaSSIF) was purchased from Biorelevant.com Ltd., UK. Transcutol P was gifted from Gattefossé, France, Softisan 601 was gifted from 101 Oleochemical Pharma, Germany, and Kolliphor RH40 was received as a gift from BASF, Germany. Carbopol^®^ Ultrez 10 (Ultrez 10) was gifted from The Lubrizol Corporation, USA. Deionised (DI) water was obtained from the Elga PURELAB system.

### Bioactivity of lacticin 3147 against *S. aureus* DMSZ20231

An overnight culture of *S. aureus* DMSZ20231 in BHI broth grown at 37 °C with shaking (70 rpm) was diluted to an optical density (OD) of 0.1 @ 595 nm. Due to lacticin 3147’s hydrophobicity, the lyophilised lacticin 3147 peptides were dissolved separately at 1 mg/ml in 70% IPA to ensure they were in solution (and filtered through 0.2-μm polyethersulfone (PES) filters). The 1 mg/ml solutions were then diluted in PBS to make 100 μg/ml peptide solutions. The lacticin 3147 peptides were added in triplicate to a 96-well plate (previously washed with 0.1% BSA) to allow for end concentrations from 5.29 μg/ml Ltnα (1600 nM) and 4.56 μg/ml (1600 nM) Ltnβ to 13.23 μg/ml Ltnα (4000 nM) and 11.39 μg/ml Ltnβ (4000 nM). PBS was added to bring the well volume up to 50 μl, and then 150 μl *S. aureus* was added. The appropriate blanks and controls were set up in triplicate ensuring the addition of PBS containing 70% IPA did not affect the growth of the bacterial control. The 96-well plate was then incubated in a microplate reader (BioTek ELx808 Ultra) at 37 °C for 24 h. Readings were taken every 30 min @ 595 nm with mild shaking before each reading. The effect of UV light on lacticin 3147’s activity was then investigated. The experiment described above was repeated for the two highest concentrations of lacticin 3147 with exposure to UV light for 30 min after their addition to the 24-well plate, and before the addition of PBS and bacteria.

### SLN production and characterisation

SLNs were fabricated as previously optimised by Ryan et al. with minor changes [11]. For SLNαβ, 5 mg of both Ltnα and Ltnβ were weighed into the same glass vial, resuspended in 80 μl DMSO and stirred at 37 °C for 15 min to ensure complete dissolution. Water was heated (5 ml) at 55 °C. A lipid-surfactant-cosurfactant solution was prepared as followed: Softisan 601 (100 mg), Kolliphor RH40 (100 mg) and Transcutol P (50 mg) were weighed out into the same vial and heated at 55 °C until dissolved and the vial was placed briefly on a stirrer plate to achieve a homogeneous solution. The lacticin 3147 solution was then added to the lipid-surfactant-cosurfactant solution and stirred briefly before heating again. Finally, the 5 ml of water (previously heated) was quickly added to the lipid-lacticin 3147 solution under stirring at 1700 rpm and remained stirring until it reached room temperature. The dispersion was probe sonicated (jacketed with ice) with a Qsonica CL-18 4422 sonicator probe at 40% amplitude, 15 s on and 15 s off for 10 min. An aliquot was taken at 0 and 24 h, diluted in water and analysed by dynamic light scattering (DLS) using the Malvern nanozetasizer to determine the size and polydispersity index (PDI) of the dispersion. Blank SLNs were also produced with just DMSO added to the lipid-surfactant-cosurfactant solution. The zeta potential of the dispersion was measured at 0 h using the nanozetasizer. The encapsulation efficiency was determined as described by Ryan et al. [12]*.*

Scanning electron microscopy (SEM) images of *L. monocytogenes ATCC1916* treated with lacticin 3147, free (freeαβ) and encapsulated in the SLNs (SLNαβ), were taken on the Hitachi SU-70 system. *S. aureus* (990 μl, log phase) was incubated separately with 10 μl PBS, 10 μg/ml (10 μl) of a 1 mg/ml free lacticin 3147 aqueous solution, 10 μl blank SLNs and 10 μg/ml (10 μl) of a 1 mg/ml lacticin 3147 SLN dispersion for 12 h at 37 °C with shaking at 70 rpm. Following incubation, the cells were separated from the supernatant via centrifugation (1500 rpm for 3 min). The post-treated cells were fixed with 2.5% glutaraldehyde in PBS, washed with PBS once and sterile water thrice with centrifugation (1500 rpm for 3 minu) and freeze-dried in a Telstar Lyoquest freeze dryer at − 80 °C and 0 mbar. A thin layer of fixed and dried cells was spread onto double-sided conductive carbon tape glued over a metallic stub and coated with gold for 120 s at 20 mA using an Emitech K55 system. This was also repeated for *L. monocytogenes,* with 999 μl of the bacteria (log phase) incubated separately with 1 μl PBS, 1 μg/ml (1 μl) of a 1 mg/ml free lacticin 3147 aqueous solution, 1 μl blank SLNs and 1 μg/ml (1 μl) of a 1 mg/ml lacticin 3147 SLN dispersion for 12 h at 37 °C with shaking at 70 rpm.

The activity of the lacticin 3147 SLN dispersions versus *S. aureus* DMSZ20231 was determined via total plate counts by the single drop method [65]. SLNαβ (1 mg/ml) was filtered through a 0.2-μm PES filter, then added to a 24-well plate (previously washed with 0.1% BSA) at volumes ranging from 125 to 7.8 μl to allow for end concentrations of 250–15.6 μg/ml lacticin 3147. Sterile water was added to make the volume up to 125 μl. *S. aureus* (OD 0.1 @595 nm) was then added to make the volume up to 500 μl and the plate was incubated for 3 h at 37 °C with shaking at 70 rpm. The samples were then diluted, plated on TSA 6 g/L yeast and left dry, and the plates were incubated overnight at 37 °C. This was repeated for blank SLNs (125 μl blank SLNs and 375 μl *S. aureus*) and the bacterial control (125 μl water and 375 μl *S. aureus*). The colony-forming units (CFU) were counted and the log(CFU/ml) and log reductions compared to the bacterial control were calculated.

### Gel optimisation

For the preparation of a topical SLN gel, two gelling agents, HPMC and Ultrez 10, were trialled at different % w/w with water. For the HPMC gels, HPMC powder was added slowly to a beaker of water (at 0.7, 1.0, 2.0, 3.0% w/w) under stirring at room temperature. The solution was stirred and monitored until gel formation was observed. For Ultrez 10 gels, Ultrez 10 powder was added slowly to a beaker of water (at 0.7 and 1.0% w/w) under stirring. The solution was then stirred at 450 rpm until the powder had fully dissolved (3.5 h). After such time, TEA (50%) was added to the solution at 0.4% v/v. Once the gel began to form, the beaker was removed from the stirring plate and the gel was stirred using a glass rod to ensure homogeneity.

### SLN gel production and characterisation

Blank SLNs and SLNαβ were produced as outlined in the section ‘SLN production and characterisation’. They were let stand at room temperature for 25 min, then filter sterilised through a 0.2-μm PES filter. To ensure the gel was made aseptically, the Ultrez 10 powder was sterilised under UV light for 30 min before use. The sterile Ultrez 10 powder (1.0% w/w) was added to the dispersion slowly while stirring and left stir at 450 rpm for 3.5 h. Subsequently, 0.4% v/v filter sterilised 50% TEA was added. The gel was mixed with a glass rod and was subsequently characterised as follows. The pH of the gel was determined using pH strips. The spreadability was investigated using a method previously described in the literature [27]; using a spatula, 0.5 g of gel was placed inside a premarked 1-cm diameter circle on a glass plate. A second glass plate was placed gently over the gel and a 500 g weight was placed on top of the upper glass plate for 5 min. After such time, the increase in diameter was recorded. The viscosity of the blank SLN gel was measured using a Brookfield DV3T rheometer. The viscosity at 50 rpm (torque 78.5%) was recorded. Finally, the particle size of the SLNs after incorporation into the gel was determined as follows: the SLN gel (0.1 g) was weighed into a vial, water was added (5 ml) and it was vortexed until the gel fully dissolved. The solution was filtered through a 0.45-μm PES filter before analysis by DLS on the Malvern Zetasizer. For the production of free lacticin 3147 hydrogel (freeαβ hydrogel), the same gel production method was used with 5 ml of a free lacticin 3147 aqueous solution (freeαβ aqueous) as the starting solution for the gel instead of the SLN suspension. Freeαβ aqueous was prepared by weighing out dried lacticin 3147 peptides and adding water to make a 1 mg/ml solution which was stirred for 5 min and let stand for 25 min at room temperature, then filter sterilised through a 0.2-µm PES filter.

The gels were stored at 4 °C and were used within 5 days for all experiments reported, with no detectable loss in activity.

### Release of lacticin 3147 from an SLNαβ gel, freeαβ hydrogel and freeαβ aqueous solution

SLNαβ gel, freeαβ hydrogel and freeαβ aqueous were prepared as previously described. To ensure the same theoretical weight of lacticin 3147 is added (if all was dissolved) in all trials, the extra weight of the SLNs and the gel components in the SLNαβ gel must be accounted for. Thus, different amounts of each sample were used in the assay. Sterile SLNαβ gel (0.700 g), freeαβ hydrogel (0.665 g) and freeαβ aqueous (0.662 g) were added to separate dialysis tubes (molecular weight cut off of 6 kDa) previously washed with 70% EtOH and sterile water. Fasted state simulated intestinal fluid (FaSSIF) was made up according to the manufacturer’s instructions (Biorelevant Ltd.) and filter sterilised through a 0.2-μm PES filter. Sterile FaSSIF (4.5 ml) and a sterile stirrer bar were added to a sterile glass vial. The dialysis tube was added to the same vial and the top was sealed with UV-sterilised parafilm. The vials were incubated at 37 °C and stirred at 360 rpm. At 3, 24, 48, 72 h, etc., the FaSSIF release medium was fully removed and replaced with fresh FaSSIF. The release medium (100 μl) was then incubated with 100 μl of *L. monocytogenes* (OD 0.1 @595 nm) in a 96-well plate for 3 h at 37 °C and shaking at 70 rpm. Following incubation, the samples were diluted in PBS and plated on TSA supplemented with 6 g/L yeast extract. The CFU were counted and the CFU/ml and log(CFU/ml) were subsequently calculated. The release medium was removed, tested and replenished every 24 h until activity ceased. For the calculation of the significant difference of the lacticin SLN gel release media at each time point versus the bacterial control, the log(CFU/ml) of the bacterial control and the lacticin SLN gels at that time point was used. The control shown on the graph is an average of all of the controls determined at each time point throughout the study for simplicity (Fig. 7).

### Ex vivo activity of SLNαβ gel and freeαβ hydrogel

Pig skin was received and used within 48 h of culling. The skin was shaved, cleaned and cut into 5 cm^2^ sections. It was first soaked face down in 70% ethanol to sanitise the skin. After this, it was dried under airflow in a biological safety cabinet and then exposed to UV light for 1 h. Once dry, 0.08 g (100 μl) of fully grown *S. aureus* Xen-29 (bioluminescent) culture was applied to the skin and allowed to establish for 30 min. Bioluminescent images were taken on the in vivo imaging system (IVIS). 0.24 g of SLNαβ gel, 0.23 g of freeαβ hydrogel and 0.24 g of blank hydrogel were applied separately to the skin with sterile spatula to help spread gels uniformly. The skin was incubated at 37 °C for 1 h in the dark before retaking the bioluminescent images.

### Statistical analysis

Standard deviations were determined using GraphPad Prism version 8.0.1. Analysis of variance (ANOVA) was utilised when required for the determination of variance and thus statistical significance of samples. ANOVA was carried out using the ‘Analyze Data’ tool in the ‘Analysis’ tab in GraphPad Prism version 8.0.1. An alpha value (α) of 0.05 was used for ANOVA, and each analysis was carried out at least in triplicate.

## Results and discussion

### Bioactivity of lacticin 3147 against *S. aureus* 20231

**Fig. 1:**
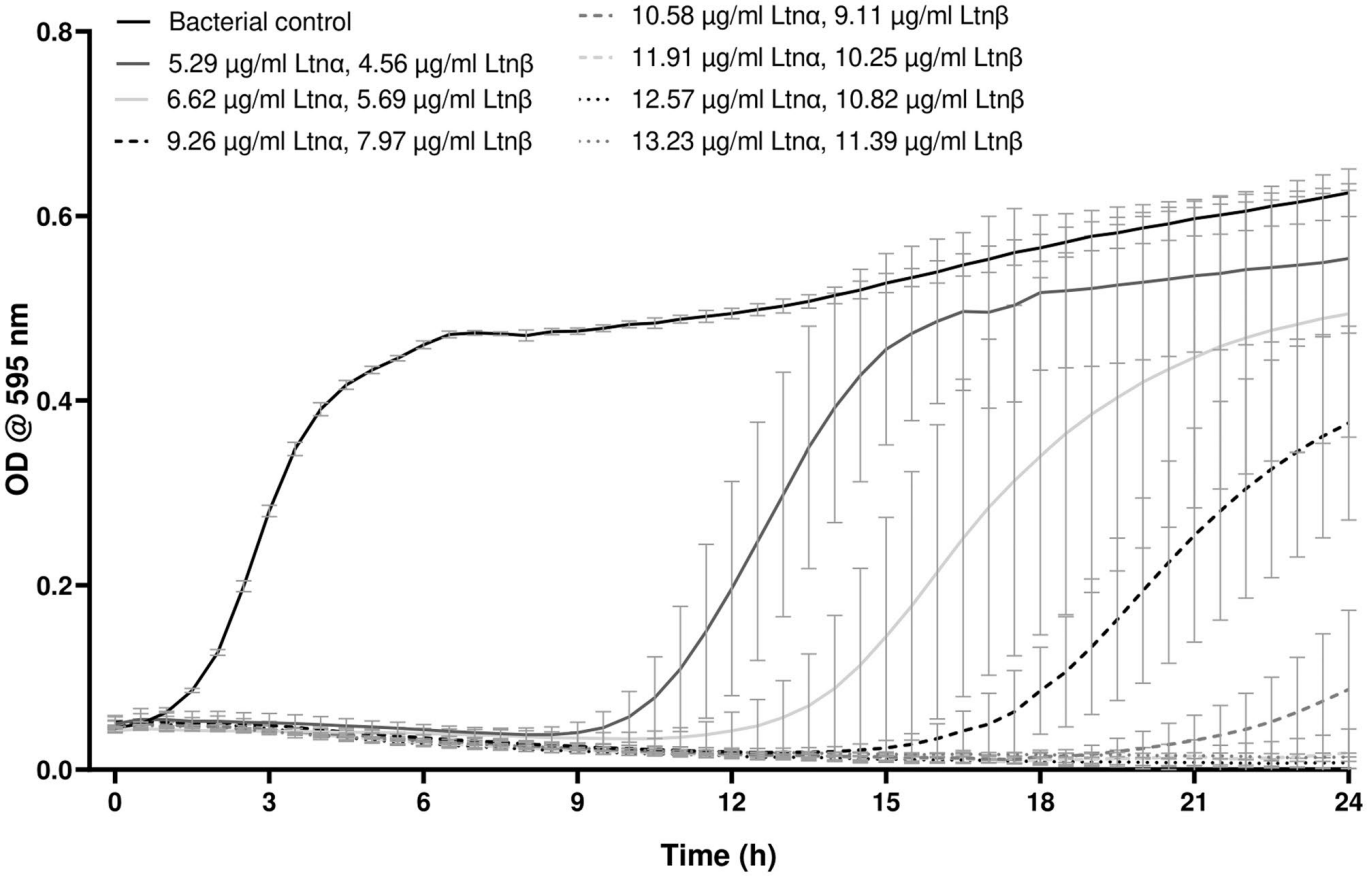
The bioactivity of lacticin 3147 against *S. aureus* 20,231 where the peptides were initially dissolved in 70% IPA, filtered then diluted in PBS, *n* = 3, mean ± SD

As *S. aureus* infections are one of the most common wound infections [20], the bioactivity of lacticin 3147 against this bacterium was first established. This was then used to determine the optimum concentration of lacticin 3147 to incorporate into SLNαβ gel and freeαβ hydrogel (Fig. 1).

As determined via growth curve assays, lacticin 3147 fully inhibited *S. aureus* DMSZ20231 at concentrations as low as 11.91 μg/ml Ltnα and 10.25 μg/ml Ltnβ (3600 nM) (Fig. 1) which is within the concentration range recorded in the literature that resulted in full inhibition of *S. aureus* strains, 1.9—15.4 μg/ml [24]. The value obtained in this study is 12 times more concentrated than that reported to fully inhibit *L. monocytogenes* ATCC1916 (0.99 μg/ml Ltnα and 0.85 μg/ml Ltnβ [11]. Thus, to achieve loadings of lacticin 3147 in the gels that would demonstrate a sustained activity against *S. aureus*, 1 mg/ml of the lacticin 3147 peptides in the SLNαβ dispersions (5 times the previous concentration of lacticin 3147 in the SLN suspensions produced by Ryan et al*.* [12]) was produced and their activity against *S. aureus* was investigated before formulating into gels.

**Fig. 2:**
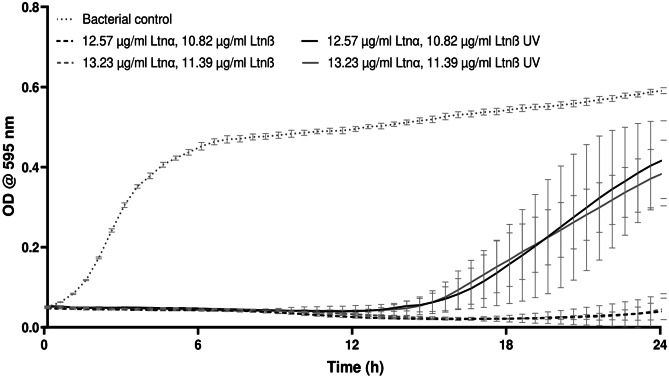
The effect of UV light on the bioactivity of lacticin 3147 against *S. aureus* 20,231 when dissolved in 70% IPA, filtered then diluted in PBS, *n* = 3, mean ± SD

Another important parameter that needed to be established before the formulation of SLNαβ gel and freeαβ hydrogel was how to ensure the sterility of the gels. The first choice was to expose the gels to UV light for 30 min after production as terminal sterilisation is often the preferred industrial method. To this end, the effect of UV exposure on lacticin 3147’s activity was first investigated (Fig. 2).

No bacterial growth occurred within 24 h when the non-UV exposed lacticin 3147 solutions were incubated with *S. aureus* (Fig. 2). When the same lacticin 3147 solutions that have been exposed to UV light were incubated with *S. aureus*, however, an increase in bacterial growth (≈ 25% inhibition) was evident; thus, exposure to UV light reduces the activity of lacticin 3147. Although many bacteriocins have been reported to be stable after exposure to UV light in the literature [66–70], it is clear from Fig. 2 that lacticin 3147 is unstable after exposure to such. It was concluded that exposure to UV light is not a suitable means for the sterilisation of the SLNαβ gel and freeαβ hydrogel. Instead, the production of the gels under aseptic conditions using sterile filtration methods for lacticin 3147 solutions and suspensions was deemed optimal/suitable.

### Lacticin 3147 SLN dispersion (1 mg/ml) production and characterisation

**Fig. 3:**
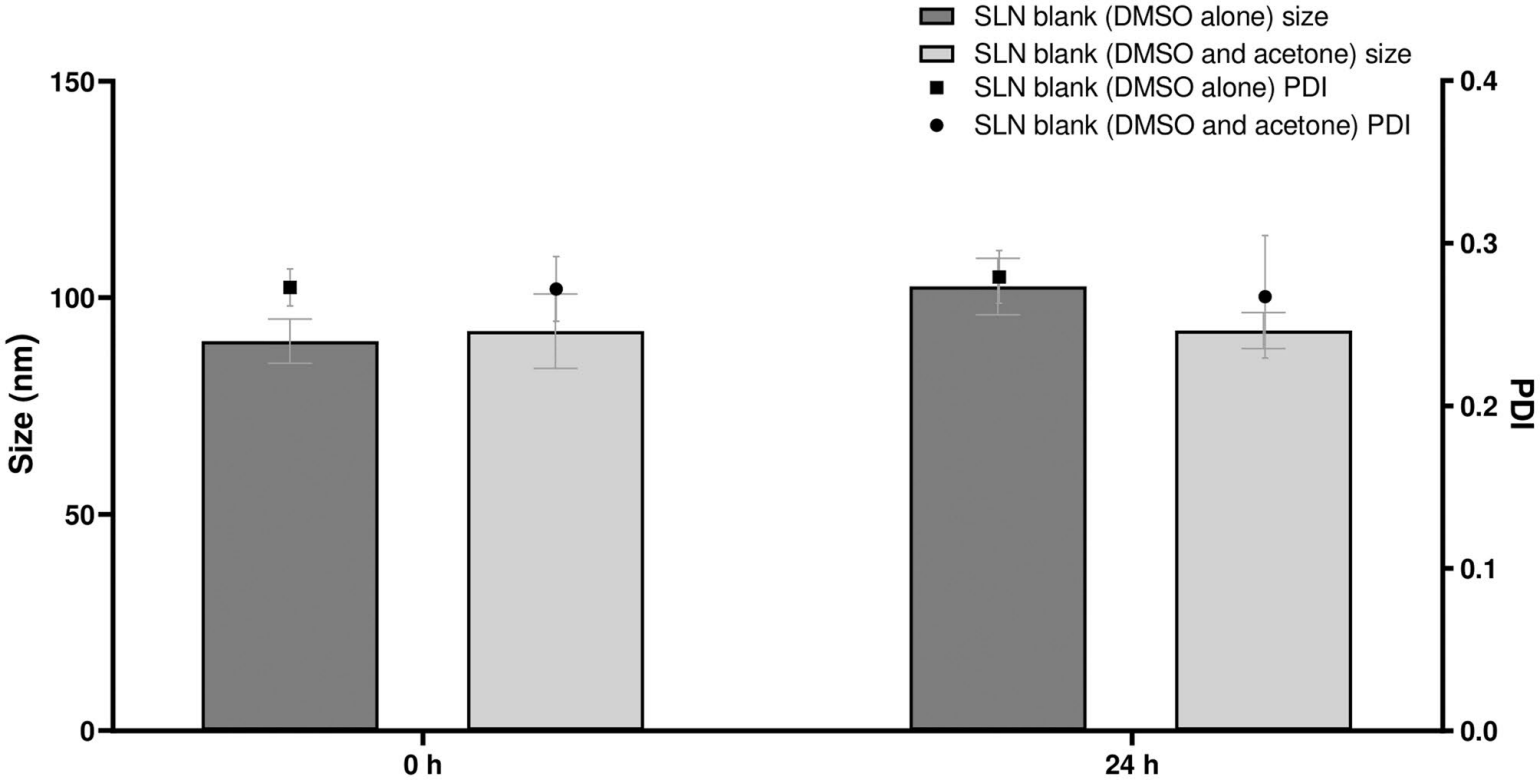
The size and PDI of the blank SLN dispersions produced with DMSO alone versus the blank SLN dispersions produced with DMSO and acetone, *n* = 10, mean ± SD

Minor changes were made to the SLN production method used by Ryan et al. [12]. DMSO alone was used to dissolve the lacticin 3147 peptides instead of DMSO and acetone. This helped with the solubilisation of the peptides and removed the 5-h stirring step previously required. Thus, the size and PDI of the blank SLNs were determined by DLS to ensure this minor change in the production method did not affect the size and PDI of the SLN dispersions produced (Fig. 3).

There is no significant difference between the size and PDI of the blank SLN dispersions made with DMSO alone and with DMSO and acetone (Fig. 3). Therefore this minor change in the SLN production method did not affect the SLN size and/or homogeneity of the SLN dispersions.

**Fig. 4:**
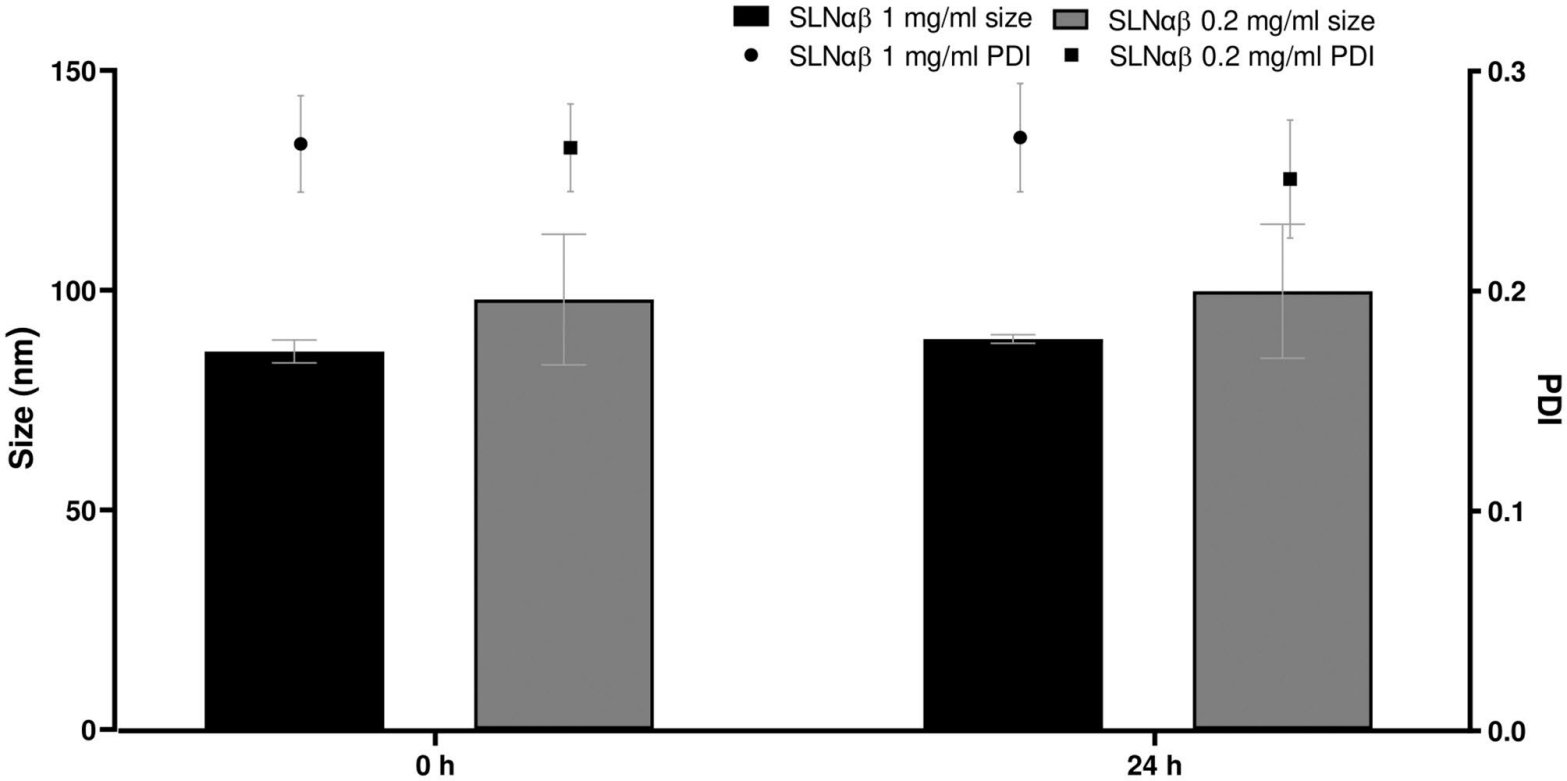
The size and PDI of SLNαβ 1 mg/ml dispersion versus SLNαβ 0.2 mg/ml dispersion, *n* = 10, mean ± SD

The same SLN production method was used here to make the 1 mg/ml lacticin 3147-loaded SLN dispersions as was used by Ryan et al. [12], to produce 0.2 mg/ml lacticin 3147 SLN dispersions (the concentrations here refer to the concentration of lacticin 3147 in the SLN suspension). Thus, the size and PDI of the 1 mg/ml lacticin 3147 SLN dispersion were determined by DLS (Fig. 4) to ensure the stability of the SLN dispersion is maintained despite the increased concentration of lacticin 3147 incorporated into the SLN dispersion.

No significant difference was observed between the size and PDI of the 1 mg/ml lacticin 3147 SLN dispersion and the 0.2 mg/ml lacticin 3147 SLN dispersion (Fig. 4). Thus, the increase in lacticin 3147 incorporated here did not affect the stability of the SLN dispersion and the dispersions were stable over 24 h.

The effect of the increased concentration of lacticin 3147 incorporated into the SLN dispersion on the zeta potential of the dispersion was investigated (Table 2).

**Table 2:** The zeta potential of blank and lacticin 3147 SLN dispersions at 0 h, *n* = 3

	**Blank SLN**	**SLNαβ 0.2 mg/ml** [69]	**SLNαβ 1 mg/ml**
**Zeta potential (mV)**	−2.95 ± 0.44	−0.48 ± 0.18	+1.47 ± 0.22

There is an increase in zeta potential after the incorporation of more lacticin 3147 into the SLN dispersion (Table 2). This is due to the increased concentration of the cationic lacticin 3147 peptides interacting with the surface of the SLNs. It is less likely for charged particles (< − 30 mV or > + 30 mV) to aggregate due to electric repulsion [31]. This system, however, is already sterically stabilised by the non-ionic surfactant and co-surfactant in the SLN formulation. Thus, it is not relying on electrostatic forces alone to prevent aggregation.

The encapsulation efficiency of the 1 mg/ml lacticin 3147 SLN dispersion was determined next (Table 3).

**Table 3:** The encapsulation efficiency of 1 mg/ml lacticin 3147 SLN dispersion, n = 3

**Encapsulation efficiency %**	**0.2 mg/ml SLNαβ dispersion**	**1 mg/ml SLNαβ dispersion**
**Ltnα**	99 ± 1%	97 ± 1%
**Ltnβ**	91 ± 3%	95 ± 2%

There was no significant difference between the encapsulation efficiencies for Ltnα or Ltnβ when the concentration of the peptide incorporated in the dispersion was increased (Table 3). For the 1 mg/ml SLNαβ dispersion, the total peptide loadings were 96 mg of peptides per g of lipid. In terms of the mass of peptide-loaded per g of lipid, there was no significant difference between the loadings of the two peptides. As the lacticin 3147 peptides act synergistically [9, 10], an equimolar ratio of loaded peptides is desired. Despite the increased concentration of the lacticin 3147 peptides incorporated into the SLN dispersion, an almost equimolar loading (1:1.2, Ltnα:Ltnβ) was still achieved.

**Fig. 5:**
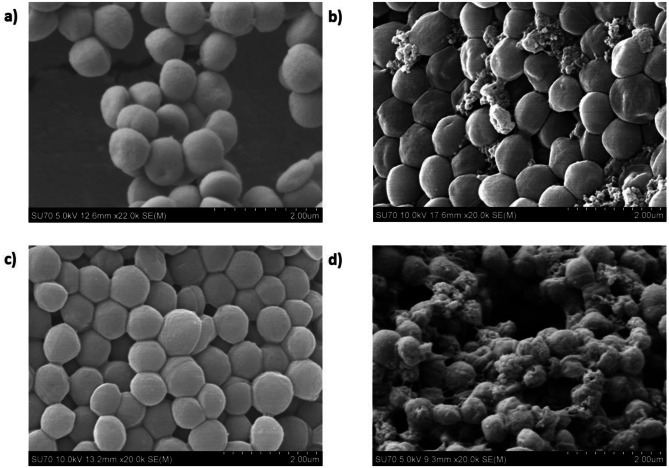
SEM images of *S. aureus* after 12 h treatment with equal volumes of **a** PBS (control), **b** a free lacticin 3147 aqueous solution (10 μg/ml lacticin 3147), **c** a blank SLN dispersion and **d** a lacticin 3147 SLN dispersion (10 μg/ml lacticin 3147), *n* = 2

SEM images were taken of *S. aureus* after exposure to the 1 mg/ml SLNαβ dispersion and an aqueous 1 mg/ml free lacticin 3147 solution (Fig. 5) at a final lacticin 3147 concentration of 10 μg/ml with the bacteria. Increased killing of *S. aureus* by 10 μg/ml SLNαβ versus 10 μg/ml free lacticin 3147 was noted. This was indicated by increased membrane damage, morphology alteration and increased leakage of cell contents in Fig. 5d compared to Fig. 5b. No major change in morphology, membrane damage or leakage of cell components was noted for blank SLNs (Fig. 5c) confirming the antimicrobial effects seen are due to the encapsulated lacticin 3147 peptides. *L. monocytogenes* is another indicator strain used for testing the antimicrobial activity of lacticin 3147. Thus, this assay was repeated with *L. monocytogenes* after exposure to a lower concentration of lacticin 3147 in the SLNs and as free peptides (1 μg/ml) due to lacticin 3147’s more potent activity against this bacterial strain. The same trend in results was observed for *L. monocytogenes* after incubation with the lacticin 3147 samples; enhanced killing is again caused by SLNαβ compared to freeαβ image (Online resource 1). Therefore, it can be concluded that SLNs enhance the antimicrobial potency of lacticin 3147. This may be due to the increased aqueous stability of lacticin 3147 when encapsulated in the SLNs. It has also been proposed in the literature that lipid nanoparticles can deliver antimicrobials close to the bacterial membrane [71–73]. The slightly positive charge of the lacticin 3147-loaded SLNs may also be increasing the affinity of the SLNs to the bacterial cells, directing bacteriocin delivery closer to its site of action [71, 73, 74]. Although more tests would be required to probe this theory, the SLNs themselves could be delivering lacticin 3147 closer to the bacterial cells thus increasing the bacteriocin’s potency.

**Fig. 6:**
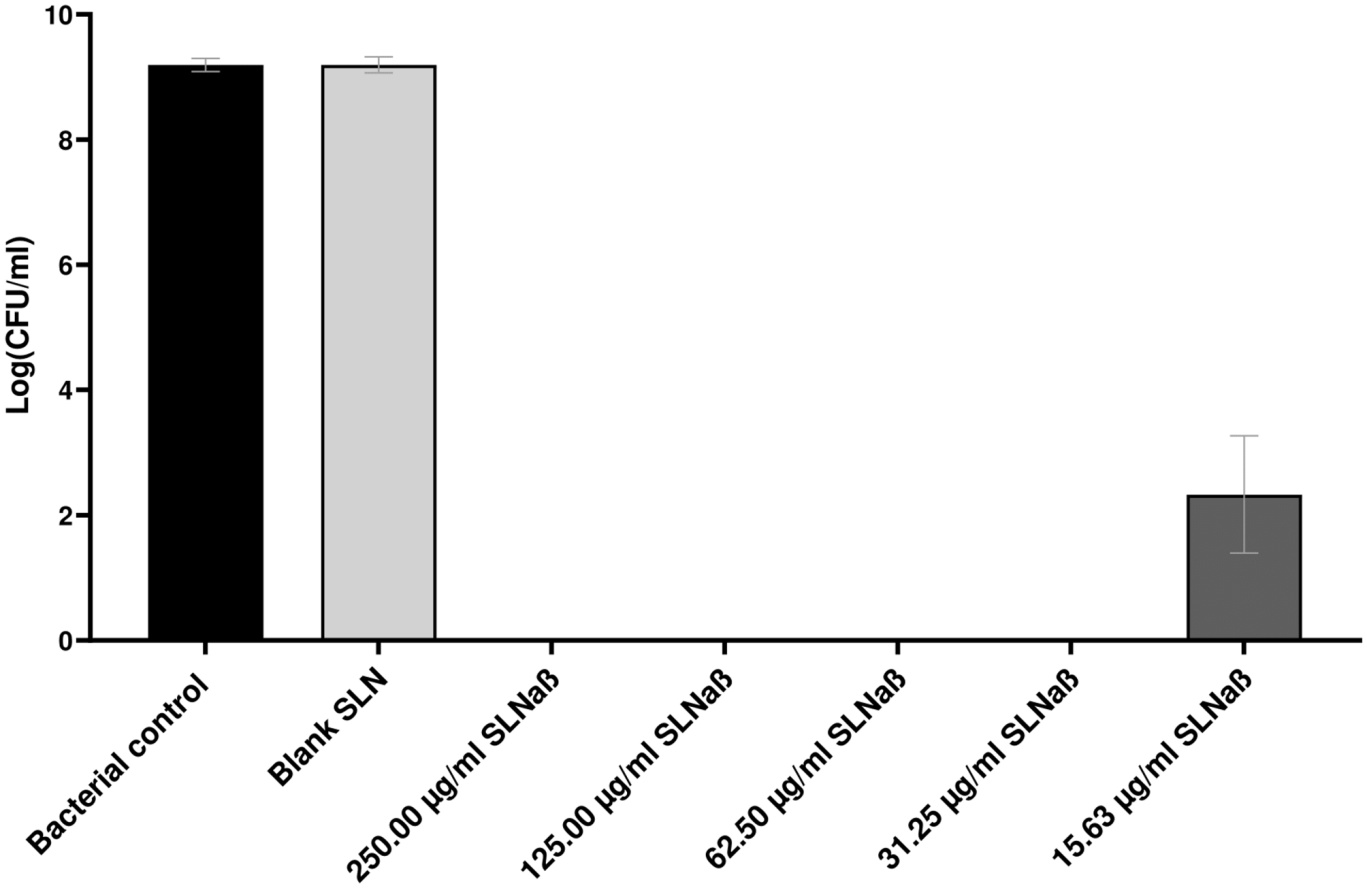
The antimicrobial activity of 1 mg/ml lacticin 3147 SLN dispersion against *S. aureus* after 3-h incubation, *n* = 3, mean ± SD

To quantify the activity of the 1 mg/ml lacticin 3147 SLN dispersion against *S. aureus*, a total plate count antibacterial assay was performed and the log(CFU/ml) for each sample was calculated (Fig. 6).

It is evident from Fig. 6 that the 1 mg/ml SLNαβ dispersion was active against *S. aureus* with full inhibition of bacterial growth from 250 to 31.25 μg/ml lacticin 3147. Although growth does begin at 15.63 μg/ml, a log reduction of 6.86 compared to the control is still seen, meaning that there was a reduction of more than 99.9999% of bacteria. There is no significant difference in bacterial growth in the bacterial control and the blank SLN dispersion; thus, the inhibitory activity of SLNαβ is a result of the encapsulated lacticin 3147 peptides.

### Gel optimisation

After the successful production and characterisation of 1 mg/ml lacticin 3147 SLN dispersion, the next step was to optimise a gel formulation for them to be incorporated into. Two gelling agents were investigated, HPMC and Ultrez 10, at increasing % w/w (starting with 0.7% w/w) until a stable, viscous, aesthetic gel with good spreadability was formed (Table 4).

**Table 4:** Gel optimisation trials, *n* = 3

**Gelling agent**	**% w/w**	**Observation**
**HPMC**		
	0.7%	No gel formation after 24 h
	1.0%	Little gel formation after 24 h
	2.0%	Free-flowing gel 4 h
	3.0%	Free-flowing gel immediately
**Ultrez 10**		
	0.7%	Good appearance but less viscous
	1.0%	Stable, viscous aesthetic gel with good spreadability

HPMC is a hydrophilic polymer which can swell into a gel when it absorbs water, i.e. a hydrogel-forming polymer [75]. Ultrez 10, however, requires a crosslinking agent like TEA (50%TEA) to convert the polymeric acid to a salt, resulting in gel formation [76]. During the trials, the % v/v of 50% TEA added was kept constant at 0.4% v/v. Water was used instead of the SLN dispersions as a base solution for these gel optimisation trials.

From the literature, it can be noted that gel formation occurs at 1.5% w/w HPMC but the gel formed has low viscosity between 600 and 900 cP [77]. This is mirrored in the 2.0% w/w HPMC results where a gel was formed but with low viscosity. Thus, a higher % w/w was trialled, 3.0% w/w, but a viscous hydrogel was still not formed (Table 4). The use of a higher molecular weight HPMC could have also been used to reach the desired viscosity or as Quiñones and Ghaly found, a hybrid Carbopol^®^-HPMC gel to overcome the issue of low viscosity with their HPMC gel [77]. Here, the Carbopol Ultrez 10 gel reached the desired viscosity without any need for the inclusion of HPMC. A stable viscous, aesthetic hydrogel with good spreadability was formed at 1.0% w/w Ultrez 10. This confirms what has been seen in the literature where various % w/w were trialled (0.5, 1 and 1.5% w/w) and 1% w/w was selected due to the favourable physicochemical properties of the gel formed [45]. Ultrez 10 is also the more suitable gelling agent of the two due to its thermosensitivity; i.e. the physicochemical properties of an Ultrez 10 gel will not change as much as a HPMC gel when exposed to a change in temperature due to the lower sensitivity of Ultrez 10 to temperature [45, 78]. Thus, this polymer, Ultrez 10 (at 1.0% w/w), and production method were used for the production of an SLN gel.

### SLN gel production and characterisation

Blank SLN gels, 1 mg/ml lacticin 3147 loaded SLN gels (SLNαβ gel) and 1 mg/ml free lacticin 3147 hydrogel (freeαβ hydrogel) were produced by the optimised gel production method, outlined previously, and were subsequently characterised. The pH, spreadability and viscosity of the blank gel and the pH and spreadability of the SLNαβ gel and freeαβ gel were investigated (Table 5). The viscosity of the blank SLN gel alone was investigated due to the large mass of the gel required for the test and the limited supply of lacticin 3147.

**Table 5:** Blank SLN gel, SLNαβ gel and freeαβ hydrogel characterisation, *n* = 3

	**pH**	**Spreadability (diameter, cm)**	**Viscosity cP @ 50 rpm, 20 °C**
**Blank SLN gel**	5 ± 0	5.3 ± 0.1	33,440 ± 2,760
**SLNαβ gel**	5 ± 0	5.3 ± 0.1	n/a
**Freeαβ hydrogel**	5 ± 0	4.7 ± 0.2	n/a

The pH of the blank SLN gels was consistently pH 5 (Table 5), which is akin to the pH of the skin (4.93 ± 0.45) [79]. This implies that the gels are stable at the pH of the skin and acceptable for application to the skin. It is important to note that the addition of the lacticin 3147 peptides in the SLNs to the gels did not have any effect on the pH of the gels. Freeαβ gels also maintained this pH. Lacticin 3147 has been reported to be stable and active, particularly at low pH [6] so the slightly acidic environment of the gels will not affect the activity of the encapsulated peptides. Good spreadability of a gel is important to ensure patient compliance and uniform application to the skin. The larger the diameter of the gel, the better the spreadability [80, 81]. The spreadability of the blank SLN gel was similar to that seen for a marketed gel (Retino A with a spreadability value of 5.8 cm [27]), thus indicating their suitability for patient use. There was no significant difference between the spreadability of the blank SLN gels and SLNαβ gels; therefore, the addition of the lacticin 3147 peptides contained within the SLNs also does not affect the spreadability of the gels. The freeαβ gel, however, had a significantly lower spreadability indicating that the presence of the SLNs in the gel may increase the spreadability of the gel. The ability of a material to resist deformation in response to stress is directly measured by its viscosity [82]. Thus, the high viscosity noted for the blank SLN gel when compared to water (1 cP [83]) indicates the gel’s ability to maintain its structure when exposed to stresses. For the retention of the gel structure when applied on the skin, a high viscosity, such as that seen here, is advantageous [84].

To ensure the retention of the SLNs’ nano size when the SLN dispersion was formulated into the gel, the size and PDI of the SLNs after incorporation into and subsequent dissolution of the gels were determined by DLS.

Table 6 demonstrates that despite increasing in size, the SLNs were retained post-gel formation. The PDI did not increase significantly post-gel formation, indicating that the size increase was uniform for all SLN nanoparticles. The size increases noted here have been reported previously, when investigated, in the literature after the incorporation of SLNs into hydrogels [42, 47] and could be attributed to the interaction of the gelling polymers, Ultrez 10 polymers in this case, with the SLNs.

**Table 6:** The size and PDI of blank SLNs before (as an SLN dispersion) and after incorporation into the gels, *n* = 3

	**Size SLN dispersion (nm)**	**PDI SLN dispersion**	**Size after incorporation into gel (nm)**	**PDI after incorporation into gel**
**SLN blank**	88.5 ± 2.9	0.268 ± 0.010	182.2 ± 18.4	0.263 ± 0.023
**SLNαβ**	82.9 ± 1.9	0.292 ± 0.017	158.0 ± 2.7	0.310 ± 0.027

### Lacticin 3147 release from an SLNαβ gel versus a freeαβ hydrogel and freeαβ aqueous solution

**Fig. 7:**
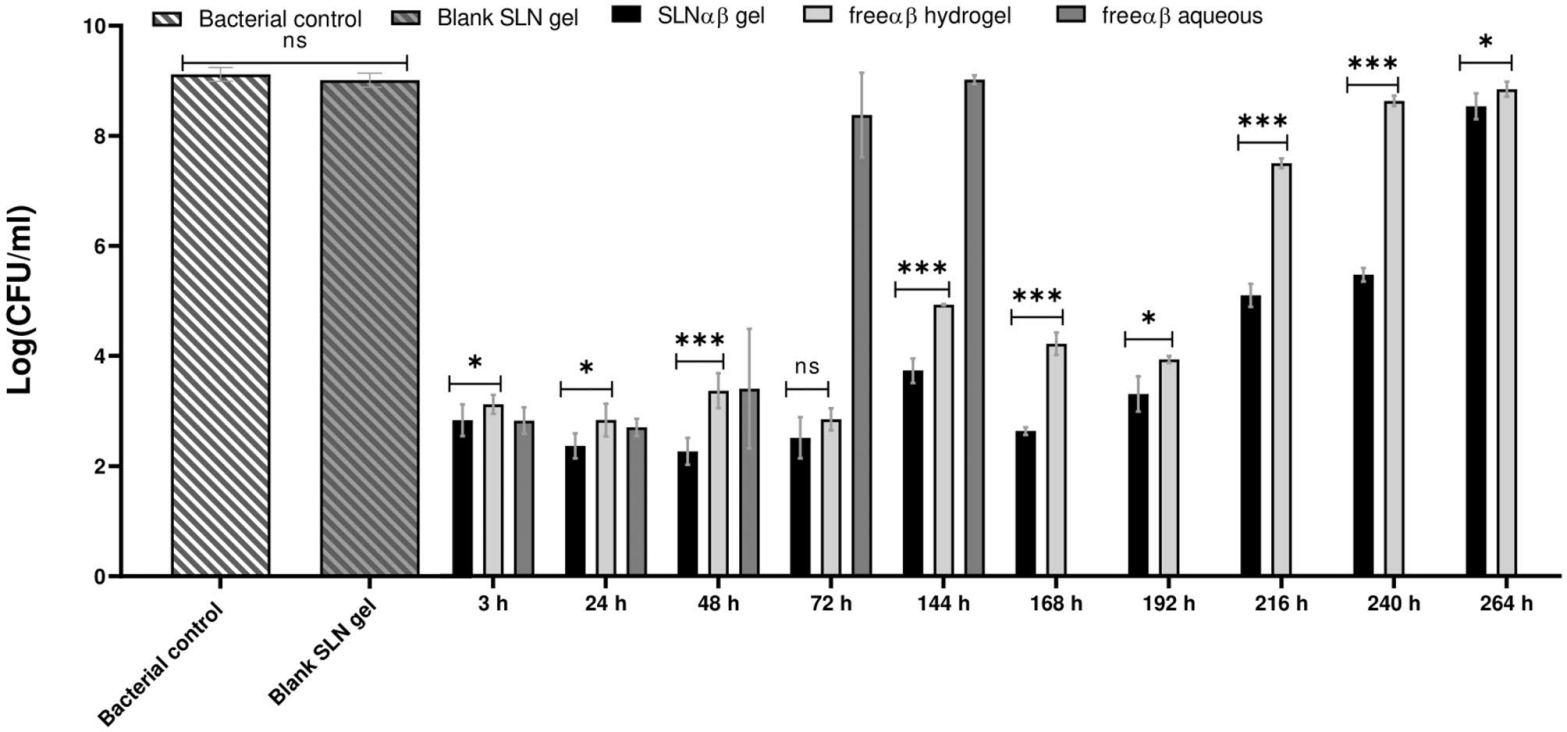
The antimicrobial activity, demonstrated by the bacterial growth (log(CFU/ml)) of L. monocytogenes after the addition of PBS as the bacterial control, shown as an average over all time points, and the growth after the addition of the release media after incubation with SLNαβ gel (active for up to 264 h), freeαβ hydrogel (active for up to 216 h), freeαβ aqueous (active for up to 48 h, not tested after 144 h) and blank SLN (inactive at all time points) over time compared to the bacterial control at each time point. *n* = 3, mean ± SD. Ns, no significant difference, one asterisk (*) = significantly different *p* < 0.05, three asterisks (***) = significantly different *p* < 0.001

Lacticin 3147 release from an SLNαβ gel, freeαβ hydrogel and freeαβ aqueous solution was inferred by measuring the antimicrobial activity of the release media (Fig. 7). An activity assay was performed in the place of HPLC analysis of the release medium due to the low solubility of lacticin 3147 in the release media. A large volume of release medium was required for the dialysis membrane used; thus, *L. monocytogenes* was used as the target bacterium for this test in the place of *S. aureus* due to its increased sensitivity to lacticin 3147.

Both the SLNαβ gel and freeαβ hydrogel release medium show prolonged activity (up to 264 h and 216 h, respectively) compared to freeαβ aqueous (up to 48 h) (Fig. 7), indicating the prolonged release capabilities of the gel formulation developed. The freeαβ aqueous solution shows similar activity to the hydrogel systems at 3 h and 24 h. At 48 h, however, a large standard deviation is present for the freeαβ aqueous solution. This is likely due to the instability of the aqueous lacticin 3147 solution. Thus, it is hypothesised that the gel formulation stabilises the lacticin 3147 peptides. Antimicrobial activity of the freeαβ aqueous solution release medium is not seen after 48 h. The SLNαβ gel release medium is significantly more active than that of the freeαβ hydrogel release medium over 264 h (11 days) at all time points except at 72 h where it shows the same activity. The freeαβ hydrogel release medium shows no activity, compared to the controls, after 9 days. This demonstrates the benefits of SLN encapsulation of lacticin 3147 within the hydrogel. No significant difference was noted between the release medium of the blank SLN gel and the bacterial control. Thus, the activity displayed by the release medium of the lacticin 3147 containing samples was due to the release of the lacticin 3147 peptides and not any other components of the systems.

### Ex vivo activity of SLNαβ gel and freeαβ hydrogel against *S. aureus* infections in pig skin

**Fig. 8:**
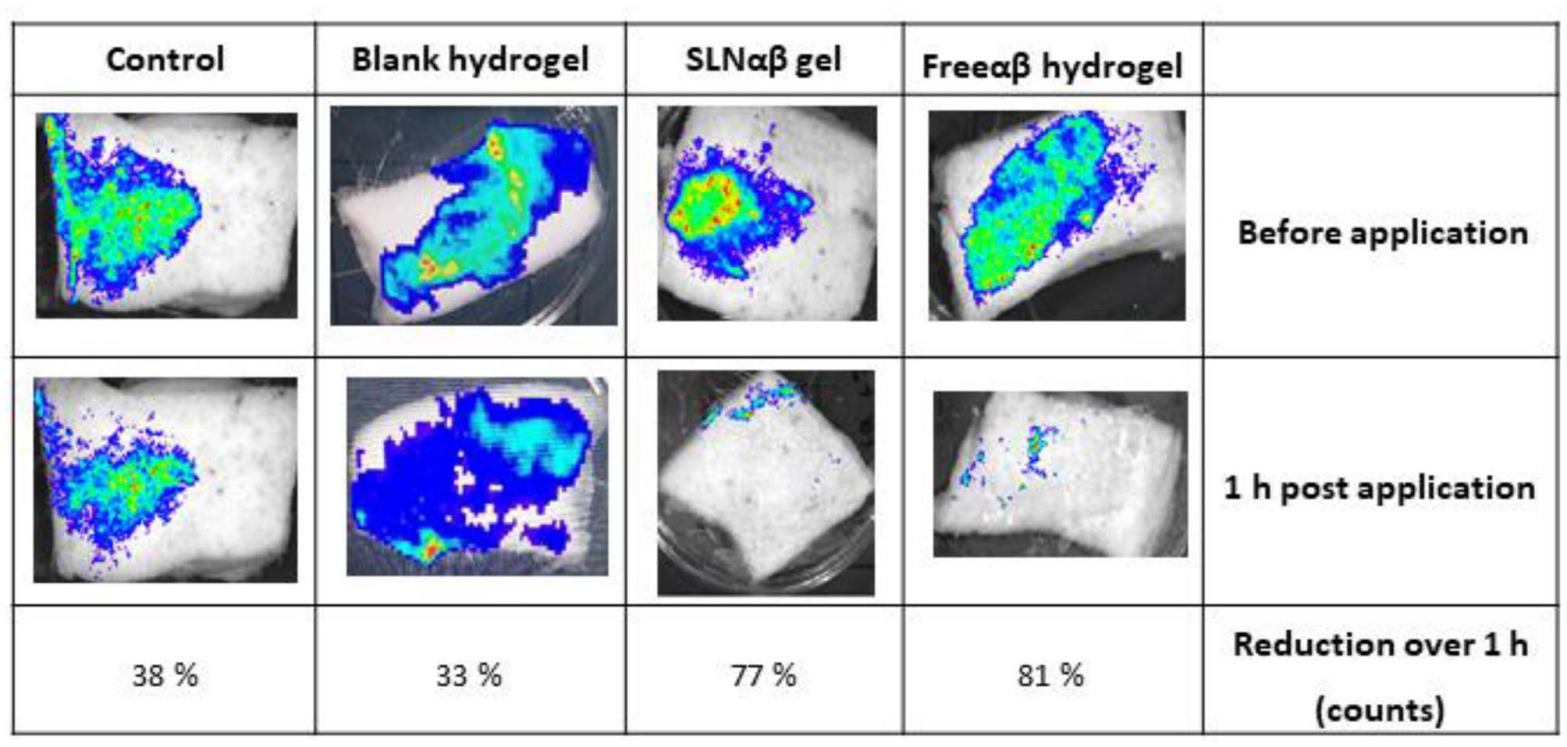
Images of segments of bioluminescently tagged *S. aureus Xen-29*-infected pig skin. The images on the top row show pig skin segments where *S. aureus* was allowed to establish for 30 min. The middle row shows the same pig skin segments after treatment with nothing (control), blank hydrogel, SLNαβ gel and freeαβ hydrogel. The bottom row shows the % reduction of bacteria over the 1 h period indicated by fluorescents counts, *n* = 3

To investigate the ability of the SLNαβ gel and freeαβ hydrogels to kill *S. aureus* infections ex vivo, both gels were applied to pig skin contaminated with fluorescent *S. aureus* (Fig. 8). Images were taken before application and 1 h after application to monitor the bacterial killing.

A large decrease in bioluminescence can be seen 1 h post application for both the SLNαβ gel and freeαβ hydrogel (Fig. 8). This indicates a significant amount of bacterial killing, demonstrating the effectiveness and potency of the lacticin 3147 gels. Little difference in fluorescence can be seen for the *S. aureus*-infected pig skin 1 h post application of blank hydrogel versus the *S. aureus* control. This confirms that it is the lacticin 3147 which causes the substantial bacterial killing, not the gel.

In this study, a gel containing lacticin 3147 SLNs was produced for topical application of lacticin 3147 to the skin. Topical drug delivery is preferred for skin and wound infections due to the reduced risk of side effects. This local delivery also ensures only the bacteria in the location of the infection are exposed to the antimicrobial thus reducing the risk of destroying commensal bacteria when delivered orally and the likelihood of resistance development in other bacteria around the body [74, 85]. For topical delivery of drugs to the skin, nanoparticles and permeation enhancers are the most recommended formulation strategy to aid the drug in crossing the main barrier of the skin, the stratum corneum [26]. Nanoparticles are recommended as their small particle size ensures close contact with the stratum corneum and acts as a drug reservoir maintaining higher drug concentration in the skin. They are preferred over permeation enhancers as they cause less skin irritation [26, 48]. As the stratum corneum is composed mostly of lipids, solid lipid nanoparticles are advantageous over polymer-based nanoparticles in achieving high drug accumulation in the skin and reduced systemic absorption when delivered topically to the skin [48, 86]. Lipids are also present in sebum in hair follicles providing another potential penetration route for dermal delivery via SLNs [26, 87]. *S. aureus* is one of the most common causes of skin and wound infections. To account for the increased concentration of peptide required to inhibit *S. aureus* compared to the previous indicator strain used, *L. monocytogenes*, an increased concentration of lacticin 3147 was used in the production of SLNαβ in this study compared to that used previously by Ryan et al. [12]. The increase in encapsulated lacticin 3147 did not cause any change in particle size, poly-diversity index or stability of the SLN dispersion. The incorporation of SLNs into suitable gel carriers ensures prolonged contact of the SLNs with the skin, thus enhancing their ability to deliver the drug into the skin layer [26]. Thus, an SLN gel formulation was optimised and characterised yielding a stable, viscous aesthetic gel with good spreadability suitable for topical application to the skin. The physicochemical properties of the SLN gel were not altered by the encapsulated lacticin 3147. The size of the SLNs increased uniformly after their incorporation into the gel system. It was proposed that this may be due to the interaction of the Ultrez 10 polymers with the surface of the SLNs.

A release study showed that the formulation of lacticin 3147 into gels enhanced the killing causing a five-fold reduction in bacterial growth at 72 h onwards, displaying the sustained release effect the gels impart. The free lacticin 3147 hydrogel achieved sustained release for 216 h (9 days) as shown by the activity of the release media. This is similar to the release period observed for bacteriocins formulated into gels in the literature: Garvicin KS released for 8 days (by HPLC) when formulated into a hybrid gel for topical delivery [16]. Nisin A was found to release from an injectable hydrogel for 10 days, although the log reductions caused by its release were much smaller than those noted here throughout the time frame of the study [88].

The SLNαβ gel release medium was more active than the freeαβ hydrogel or freeαβ aqueous solution release medium over 264 h for all time points but 72 h. Thus, the SLNαβ gel system was the superior formulation, resulting in a more active, longer-acting gel. A similar trend can be noted in the literature where the release of the small molecule drug silybin from an SLN gel versus the free drug was monitored. Although the test was performed over a much shorter time period (24 h), a more prolonged release profile was still documented for an SLN silybin gel (24 h) compared to free silybin (6 h). It is important to note that a different SLN composition (Precirol ATO5, Tween 20 and Span 80) and gelling polymer (Carbopol 940) was used in the silybin study. Thus, the longer release profile achieved for the SLNαβ gel shows the advantage of the specific SLN and gel formulations used in this paper compared to the silybin study. When the release of silybin from an SLN silybin gel and a free silybin gel was compared, the SLN gel also had a more sustained release profile [39]. This confirmed the advantages of the SLNs in gel systems.

Both the SLN and free lacticin 3147 gels showed potent activity against *S. aureus*-contaminated pig skin, with significant bacterial eradication (> 75%) after 1 h of infection. This displayed their effectiveness ex vivo. The results achieved in this study indicate the potential of a lacticin 3147 SLN gel as a topical treatment for wound infections. Further animal studies, such as the effect of the gel on in vivo mice infected wound model [16], would be required to investigate the ability to translate these results to a therapeutic dose and whether the lacticin 3147 SLN-gel formulation would be clinically different to the free peptide gel.

## Conclusion

A multi-hurdle approach to the development of new antimicrobial treatments has been recommended by the WHO to prevent the emergence of antimicrobial resistance to the treatment. This approach is not ideal due to the expenses involved and the potential for more severe side effects. In this study, the delivery vehicle itself, the SLNs, and the antimicrobial, lacticin 3147, act synergistically, enhancing the bacterial killing for prolonged periods of time. For application to the skin, a gel containing the lacticin 3147 SLNs (SLNαβ gel) that displayed sustained release for 11 days and was active against a *S. aureus* infection on skin was developed. This long-acting potent SLNαβ gel has the potential to be used as a topical treatment of *S. aureus*-infected wounds if these results can be mirrored in an in vivo mice wound model.

## Supplementary Information

Below is the link to the electronic supplementary material.

Supplementary file1 (PDF 1008 kb)

## Data Availability

All data and material is available on request from the corresponding author.

## Declarations

### Authors’ information (optional)

All enquiries can be directed to the corresponding author, Sarah Hudson, Associate Professor in Chemistry at the University of Limerick. Email address: Sarah.Hudson@ul.ie.
