# Supplementary material for: The development of a solid lipid nanoparticle (SLN)-based lacticin 3147 hydrogel for the treatment of wound infections
Source: Drug Deliv Transl Res. 2023 Mar 24;13(9):2407–23. doi: 10.1007/s13346-023-01332-9 (PMC10382363; doi:10.1007/s13346-023-01332-9)
Supplement: Supplementary file 1 — Supplementary file1 (PDF 1008 kb) [file 13346_2023_1332_MOESM1_ESM.pdf]

The development of a solid lipid nanoparticle (SLN) based lacticin 3147 hydrogel for the treatment of wound infections

Aoibhín Ryan[a], Pratikkumar Patel[a], Poonam Ratrey[a], Paula M. O'Connor[b,c], Julie O'Sullivan[c,d] R. Paul Ross[c,d], Colin Hill[c,d] and Sarah P. Hudson[a,e*].

[a]Department of Chemical Sciences, Bernal Institute, University of Limerick, Ireland,

[b]Teagasc Food Research Centre, Moorepark, Fermoy, Co. Cork, Ireland,

[c]APC Microbiome Ireland, Cork, Ireland,

[d]School of Microbiology, University College Cork, College Road, Cork, Ireland.

[e]SSPC the SFI Research Centre for Pharmaceuticals, University of Limerick, Ireland.

*Corresponding Author: Prof. Sarah Hudson, Associate Professor in Chemistry

Email: Sarah.Hudson@ul.ie, telephone: +35361234981

Supplementary Information

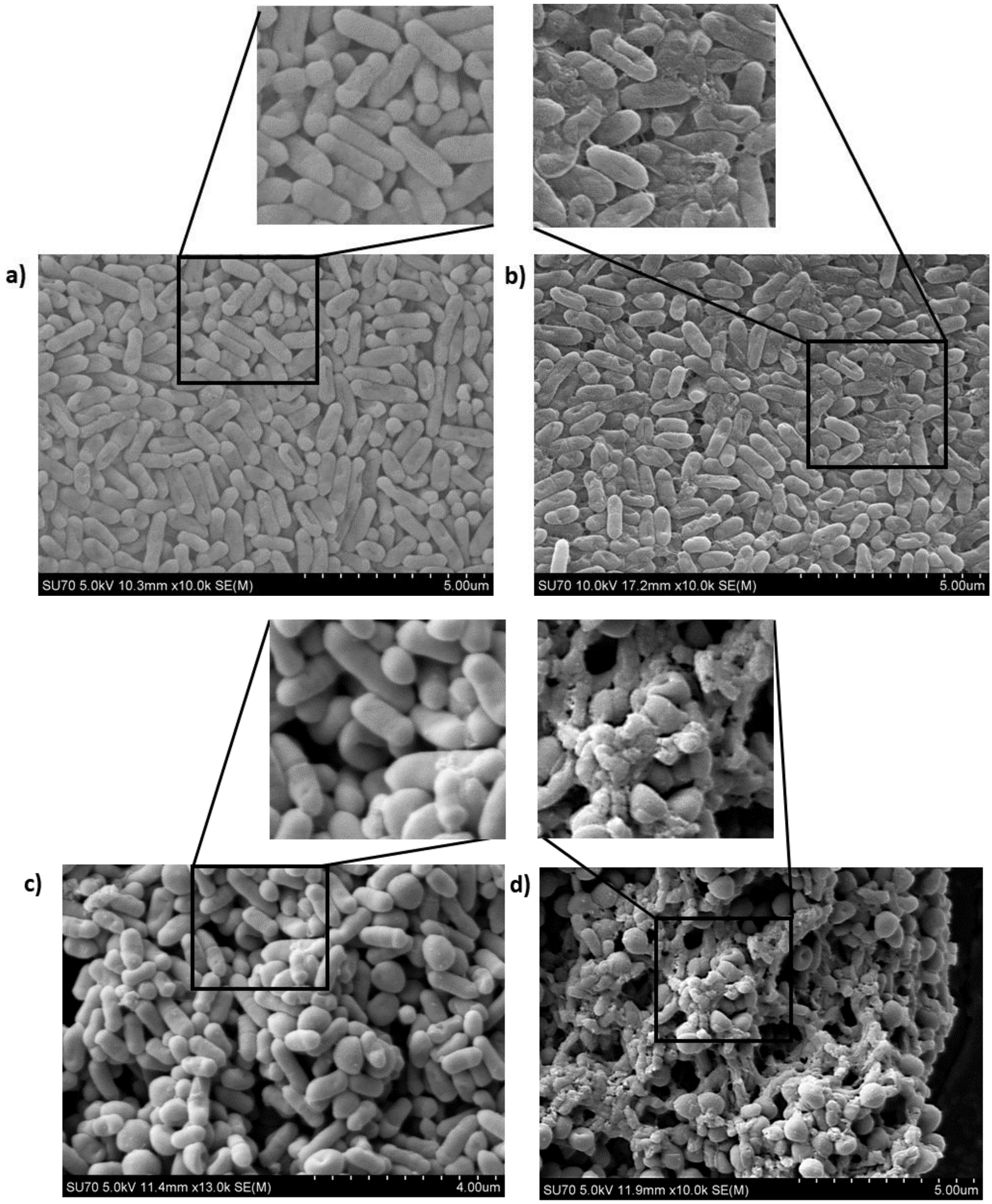

Online resource 1 SEM images of *L. monocytogenes* after 12 h treatment with equal volumes of a) PBS (control), b) a free lacticin 3147 aqueous solution (1 µg/ml lacticin 3147), c) a blank SLN dispersion and d) a lacticin 3147 SLN dispersion (1 µg/ml lacticin 3147), n=2
